# HEARING LOSS AND LONELINESS AMONG OLDER ADULTS: LIVING ALONE AS A RISK FACTOR

**DOI:** 10.1093/geroni/igae098.4202

**Published:** 2024-12-31

**Authors:** Kira Birditt, Angela Turkelson, Emily Noyer, Emma Weber, Crystal Ng

**Affiliations:** University of Michigan, Ann Arbor, Michigan, United States; University of Michigan, Ann Arbor, Michigan, United States; Survey Research Center, Ann Arbor, Michigan, United States; University of Michigan, Ann Arbor, Michigan, United States; University of Michigan, Ann Arbor, Michigan, United States

## Abstract

Hearing loss is a leading source of disability within the United States, affecting up to 65% of adults aged 71 and older in the United States. Hearing loss is associated with greater loneliness which is also common among older adults and predicts a range of negative health outcomes. However, little is known about whether hearing loss predicts changes in loneliness over time. Further, adults who live alone with hearing loss may be particularly at risk for loneliness. Using newly released longitudinal data from the Health and Retirement Study (2018 and 2022), this study seeks to examine the implications of hearing loss for loneliness over time. Participants included respondents (N = 2,753) who participated in 2018 and 2022 waves (ages 50 to 97; 60% women). Participants completed an in person hearing test and questionnaires in which they reported loneliness with the three item UCLA loneliness scale. A total of 72% of the respondents had mild to severe hearing loss and 20% lived alone. Multilevel models controlling for age, gender, race and education showed that having worse hearing predicted greater loneliness. Worse hearing predicted increased loneliness over time among older adults living alone but not those living with others. Hearing loss is an important risk factor for loneliness among older adults with those who live alone at greatest risk. Further research should examine whether using hearing aids reduces the negative implications of hearing loss for loneliness and examine factors that predict hearing aid use among older adults who live alone.

